# Opioid reduction and enhanced recovery in orthopaedic surgery (OREOS): a protocol for a feasibility randomised controlled trial in patients undergoing total knee arthroplasty

**DOI:** 10.1186/s40814-024-01457-9

**Published:** 2024-02-15

**Authors:** Kim Madden, Sushmitha Pallapothu, Darren Young Shing, Anthony Adili, Mohit Bhandari, Lisa Carlesso, Moin Khan, Ydo V. Kleinlugtenbelt, Adrijana Krsmanovic, Matilda Nowakowski, Tara Packham, Eric Romeril, Jean-Eric Tarride, Lehana Thabane, Daniel M. Tushinski, Christine Wallace, Mitchell Winemaker, Harsha Shanthanna

**Affiliations:** 1 Department of Surgery, McMaster University, Hamilton, Canada; 2 Research Institute of St. Joseph’s Healthcare Hamilton, Hamilton, Canada; 3 Department of Health Research Methods, McMaster University, Hamilton, Canada; 4 Department of Medicine, University of Ottawa, Ottawa, Canada; 5 School of Rehabilitation Science, McMaster University, Hamilton, Canada; 6 Deventer Hospital, Department of Orthopaedics, Schalkhaar, The Netherlands; 7 Hamilton Health Sciences–Juravinski Hospital, Hamilton, Canada; 8 Department of Anesthesia, McMaster University, Hamilton, Canada; 9 Center for Health Economics and Policy Analyses, McMaster University, Hamilton, Canada; 10 Department of Psychiatry and Behavioural Neurosciences, McMaster University, Hamilton, Canada

**Keywords:** Persisting pain, Feasibility, Opioid reduction, Knee arthroplasty, Multicomponent intervention

## Abstract

**Background:**

Knee arthritis is a leading cause of limited function and long-term disability in older adults. Despite a technically successful total knee arthroplasty (TKA), around 20% of patients continue to have persisting pain with reduced function, and low quality of life. Many of them continue using opioids for pain control, which puts them at risk for potential long-term adverse effects such as dependence, overdose and risk of falls. Although persisting pain and opioid use after TKA have been recognised to be important issues, individual strategies to decrease their burden have limitations and multi-component interventions, despite their potential, have not been well studied. In this study, we propose a multi-component pathway including personalized pain management, facilitated by a pain management coordinator. The objectives of this pilot trial are to evaluate feasibility (recruitment, retention, and adherence), along with opioid-free pain control at 8 weeks after TKA.

**Methods:**

This is a protocol for a multicentre pilot randomised controlled trial using a 2-arm parallel group design. Adult participants undergoing unilateral total knee arthroplasty will be considered for inclusion and randomised to control and intervention groups. Participants in the intervention group will receive support from a pain management coordinator who will facilitate a multicomponent pain management pathway including (1) preoperative education on pain and opioid use, (2) preoperative risk identification and mitigation, (3) personalized post-discharge analgesic prescriptions and (4) continued support for pain control and recovery up to 8 weeks post-op. Participants in the control group will undergo usual care. The primary outcomes of this pilot trial are to assess the feasibility of participant recruitment, retention, and adherence to the interventions, and key secondary outcomes are persisting pain and opioid use.

**Discussion:**

The results of this trial will determine the feasibility of conducting a definitive trial for the implementation of a multicomponent pain pathway to improve pain control and reduce harms using a coordinated approach, while keeping an emphasis on patient centred care and shared decision making.

**Trial registration:**

Prospectively registered in Clinicaltrials.gov (NCT04968132).

## Background

Arthritis is a very common and painful joint condition affecting 6 million Canadians, and nearly 1 in 2 Canadians over the age of 65 [1, 2]. Osteoarthritis (OA) is the most common form, affecting more people than all other forms of arthritis combined [2]. End-stage knee OA is treated by total knee replacement (also known as total knee arthroplasty; TKA), which results in substantial improvements in pain and functional outcomes for most people. TKA is the second most common surgery in Canada with > 75,000 procedures performed in Canada in 2018–2019 [3, 4]. Although TKA is considered to be a successful treatment, around 20–25% of patients have lasting pain after surgery [5, 6]. Chronic post-surgical pain (CPSP) is complex, and factors known to be associated with it include pre-operative psychological factors like anxiety, depression and pain catastrophizing; pre-existing chronic pain and opioid use; and the severity and duration of postoperative pain [7–10]. Among patients who develop CPSP after TKA, 56% of them continue use of opioid analgesics at 30 days after surgery, 40% after 4 months and 25% after 2 years [11–13]. The traditional approach to post-discharge pain management has been for orthopaedic surgeons to prescribe a set number of institutionally standardized pain management medications, which can include non-steroidal anti-inflammatory drugs (NSAIDs) and/or opioids, without accounting for individual pain trajectories and preferences. However, studies have suggested that distinguishing problematic pain resolution from normal resolution may not be possible unless we appreciate individual patterns over time by personalized assessment and management following TKA [14].

Opioids are an important part of perioperative pain management [15–17]. However, their potential for long-term adverse effects such as persistent opioid use (POU) [18], addiction and dependence, overdose, diversion of unused pills [19, 20] and death in severe cases are well recognized [15, 21]. Preoperatively, one-third of patients in Canada with end-stage knee OA use prescription opioids [22]. Based on a large database study of 69,368 arthroplasty patients, 13% of opioid-naive and 62% of chronic opioid users continued their opioid use at 1 year after TKA [23]. Patients using preoperative opioids are particularly at risk of POU; 64 to 77% of chronic opioid users continue to use opioids after surgery, particularly after arthroplasty [24, 25]. In general, reducing opioid prescriptions can certainly help as not all patients may need opioids [25–27]. However, limiting opioids without individualizing the treatment of persistent pain can potentially drive patients to illicit sources.

A recent scoping review identified 141 studies to decrease opioid use in orthopaedic surgery, of which 70 were in the arthroplasty field (49.6%). Only 8.5% (12/141) of studies followed patients beyond 7 days, only four had follow up of three or more months, further only 24% of TKA studies used multimodal interventions. None of them had a preoperative education and risk reduction component. Important findings included were (1) both preoperative pain and preoperative opioids independently increase the risk of persistent pain and chronic opioid needs [10, 23, 24, 28, 29]. Despite this, most studies have excluded such patients, thereby limiting the external validity [30]; (2) most studies are associated with attempts to achieve in-hospital opioid free care [31, 32], which has not been shown to influence long term opioid use [17]; (3) most studies have focused on single interventions with limited or no effect [33, 34]; (4) the majority of studies involve a follow-up duration of a few weeks or less [30, 31, 34]; and most importantly, (5) existing trials do not take into account the individual variability within patients for pain resolution [35].

Perioperative surgical home (PSH) care pathways are defined by the American Society of Anesthesiologists as ‘patient-centred and physician-led multidisciplinary and team-based system of coordinated care that guides the patient throughout the entire surgical experience’. Over the last decade or so, several publications have highlighted its potential role in overcoming problems at the population level by providing a system that provides coordination during all phases of surgery [36]. Despite this, a recent (2020) systematic review on PSH demonstrated only low evidence for studies supporting its use [37]. Similarly, there are no randomised controlled trials (RCTs) on transitional pain clinic approaches, which have become conceptually very popular and are currently being used in many centres [38]. Based on the literature, we identified the need of four components that form the core of our care pathway/intervention arm; (1) patient education and expectation setting, (2) identification and modification of preoperative risk factors, (3) personalized analgesic prescriptions and (4) continued support for pain control and recovery.

Before embarking on a larger trial, we plan to assess the feasibility of implementing a multicomponent pain management pathway with the use of a pain management coordinator at each site. We believe that if the results of this trial deem feasible, the definitive trial will allow for the implementation of a coordinated approach to care, to improve pain control and reduce harms, while also emphasizing patient centred care and shared decision making.

## Objectives

The principal objective is to assess the feasibility of conducting a larger randomised controlled trial (RCT) of a multicomponent care pathway versus standard care to improve pain control and decrease opioid use in TKA patients.

### Feasibility objectives

The feasibility objectives will be to evaluate adherence to the study intervention, participant recruitment and participant retention. We will observe any challenges in implementing the study interventions and data collection procedures to consider appropriate changes to the final design.

### Clinical objectives

The clinical objectives will be the objectives of the definitive trial. The primary objective for the definitive trial will be to assess the effect of the multicomponent interventional pathway on opioid free pain control at 8 weeks after TKA versus standard care. We define opioid free pain control as a state of good pain control (three consecutive days of < 4/10 average pain score on a 0–10 numerical rating scale [NRS] with no opioid use for the operated knee). Other objectives include evaluating:

- Presence of CPSP at 3, 6, 9 and 12 months [39]
- Presence of POU at 3, 6, 9 and 12 months
- Average intensity of CPSP at rest and with movement at 3, 6, 9 and 12 months
- Satisfaction with pain control at 3, 6, 9 and 12 months
- Return to function at 3, 6, 9 and 12 months
- Knee function at 3, 6, 9 and 12 months
- Quality of life at 3, 6, 9 and 12 months
- Operative and knee-related complications during the study
- Economic analyses

## Methods

### Overview of the design

**Fig. 1:**
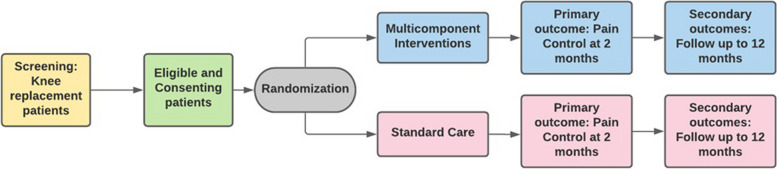
OREOS interventional pathway

This is a multicenter pilot randomised controlled trial using a 2-arm parallel group design (Fig. 1). For the pilot trial, we aim to recruit participants from three high volume arthroplasty hospitals in Ontario. If the definitive trial proceeds with no major methodological changes, we will include the pilot trial patients in the definitive trial.

### Patient selection

All patients who are being scheduled for primary elective TKA will be screened for eligibility by participating surgeons (target approximately 1–6 weeks before surgery). We will aim to include proportions of men and women in our trial that are representative of the TKA population. We will record numbers of ineligible patients and those who decline to participate. The surgeon or their delegate will inform potentially eligible patients by phone or in person to invite them to speak with the research coordinator about the trial. Each institution will determine their own recruitment processes based on local research ethics board (REB)-approved practices. Sites will be allowed to select an informed consent method that meets their REB and local institutional guidelines. This could include written informed consent or verbal consent. The informed consent process will be documented in all cases.

### Eligibility criteria

Inclusion criteria:

- Adult (18 +)
- Undergoing elective TKA for knee arthritis
- Can use a simple electronic device (phone or tablet)
- Provide informed consent to participate

Exclusion criteria:

- Revision surgery
- Simultaneous bilateral arthroplasties
- Unable to consent (e.g. cognitive disability or substantial language barrier without a support person)

### Interventions

#### Intervention group

**Fig. 2:**
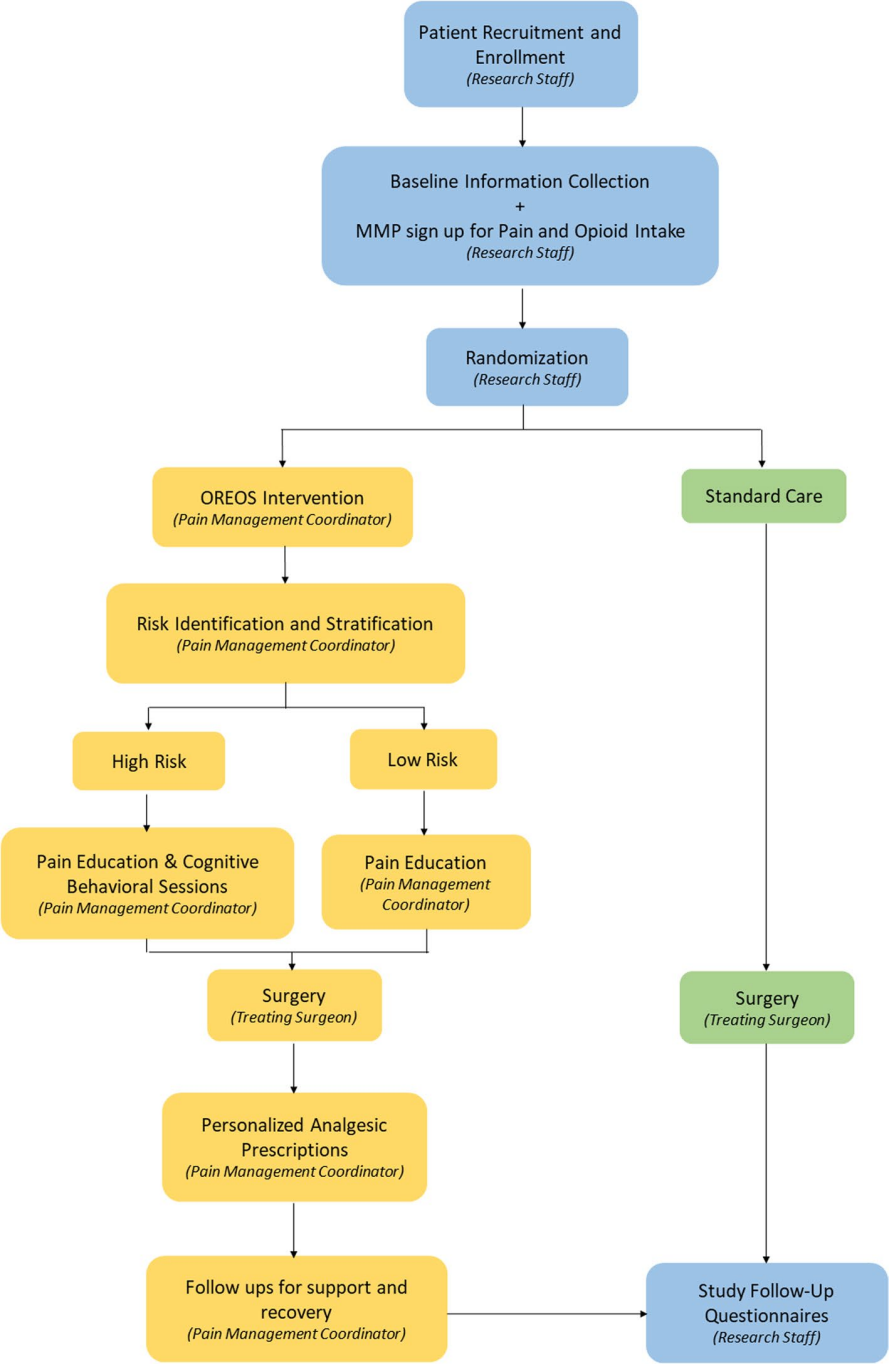
Schematic of the study intervention

Participants will participate in a multicomponent pathway coordinated by a trained pain management coordinator who will facilitate patient participation and engagement with each interventional component (Fig. 2). Study interventions will start 1–6 weeks before their surgery. In the intervention group, patients will participate in study interventions through their preoperative, in-hospital, and post-operative period, up to two months after their surgery.

Intervention participants will be stratified into high risk or standard risk based on preoperative opioid use, depression, anxiety and/or kinesiophobia by the pain management coordinator. The pain management coordinator will then facilitate the delivery of preoperative components of pain education (all intervention participants) and cognitive behavioural skill (CBS) sessions for high-risk patients (based on cognitive behavioural therapy [CBT] principles); and post-operatively, the coordinator will facilitate personalized analgesic prescriptions, and check in with patients about pain control and functional recovery. This role can be fulfilled by any health care personnel who can be trained to deliver patient education and conduct CBS sessions (e.g. medical graduate, allied health professional). All intervention components will be standardized and protocolized in an intervention manual. Study outcomes will be collected by separate research personnel not involved in the patient’s clinical care.

Pre-operatively, participants will view pre-recorded online presentations on ‘understanding pain after surgery’ and ‘managing pain after surgery’ developed by a pain physician in collaboration with a psychologist, occupational therapist and physiotherapist. Educational content includes simple pain physiology, surgical pain experience and resolution, setting expectations, goals of functional pain relief, managing daily activities and opioid benefits and risks. The coordinator will facilitate and encourage participants’ access to these online modules and will answer participants’ questions.

The pain management coordinator will conduct preoperative risk assessments based on preoperative opioid usage, depression, anxiety and kinesiophobia. Participants who meet one or more of the high risk criteria will be asked to complete two sessions of CBS sessions and suggestions on opioid sparing strategies (in-person or virtual) [7, 28, 40]. Preoperative opioid use increases the risk of poor outcomes. If the participant is considered high risk for opioid use and is willing to reduce their opioid use, the pain management coordinator will work with the site pain physician to safely reduce their opioid use. Very few opioid reduction studies focus on high-risk populations, so the evidence behind identification of individual risk components and preoperative risk education is lacking. However, this strategy is recommended by the American Society of Enhanced Recovery [28].

Post-discharge, patients will have scheduled virtual/telephone check-ins with the pain management coordinator before hospital discharge and at 1, 2, 3, 4, 6 and 8 weeks after surgery (total 7 check-ins). During these meetings, the coordinator will deliver continued support for pain control and recovery, and personalized analgesic prescriptions. The pain management coordinator will encourage the use of non-opioid analgesics and non-pharmacological measures (e.g. exercise, mindfulness, ice) [41] and encourage safe use of opioids where appropriate. The coordinator will also answer questions and facilitate virtual or in-person meetings with the surgical team if problems arise. Patients who are willing to reduce their opioid use will be supported to slowly wean their opioids, with the support of a pain physician. Based on a study assessing guided opioid tapering support, patients were able to successfully reduce/discontinue their opioid consumption following TKA [42]. Therefore, we believe that many patients will be enthusiastic about post-operative opioid tapering and discontinuation if their knee pain has been controlled by TKA.

The pain management coordinator will facilitate individualized discharge prescriptions integrating patient preferences. For example, some patients prefer not to use opioids because they have experienced adverse effects in the past, while others feel that opioids work well for them. Some patients would like to try non-pharmacological pain management strategies such as exercise or cold therapy.

#### Control group

All patients will receive usual care at their center. Presently, this does not include a pain management coordinator. Existing pre-operative knee classes at enrolling sites are not typically oriented towards pain education and appropriate opioid use. Post-operative discharge medications vary according to surgeon’s preference and are not typically individualized to patients’ needs. The research personnel will follow all participants for study outcomes.

#### Perioperative care and surgical treatment

Participants will undergo usual perioperative and surgical care at their centre. Choice of surgical technique, anesthetic technique, and in-hospital analgesia will be left to the treating surgeon, anaesthesiologist, and allied health team. Individual analgesic components in-hospital have not been shown to influence post-discharge outcomes such as POU or CPSP in larger studies [17].

### Study outcomes

#### Primary (feasibility) outcomes and criteria for success

Feasibility outcomes include intervention adherence, participant recruitment and participant retention (Table 1). We will note the percentage of participants receiving at least 3 of the 4 trial intervention components. We will consider > 90% as feasible; 80–90% to consider design modifications; and 80% as not feasible. Adherence to the intervention will be captured using an adherence checklist by the coordinator. The following criteria must be met to consider that the intervention components were adhered to for each patient: (1) patients must have reviewed both preoperative educational modules and 2 sessions of cognitive behavioural skills if in the high risk group; (2) coordinator must have stratified the patient into high or standard risk; (3) the surgeon must have provided a personalized discharge prescription, facilitated by the coordinator; and (4) the patient and coordinator must have connected in person or virtually for at least 6 of the 8 scheduled post-operative check-ins. We aim to recruit 100 patients in 4 months. We will consider > 90% participant retention to be feasible at 12 months post-operatively, 80–90% to consider design modifications and < 80% is not feasible. Table 2 shows the criteria for success of feasibility objectives.

**Table 1:** Trial objectives, outcomes and analyses

***Feasibility objectives***	***Feasibility outcomes***	***Evaluation metrics***	***Analysis***
Intervention adherence	Percentage of patients receiving at least 3 of the 4 trial intervention components	> 90% feasible; 80–90% to consider design modifications; < 80% not feasible	Descriptive
Patient recruitment	Time to recruit target sample size	We aim to recruit 100 patients in 4 months	
Patient retention	Percentage of follow up at 12 months	> 90% feasible; 80–90%-consider design modifications; < 80%-not feasible	
***Clinical Objectives***	***Clinical outcomes***		***Analysis***
Opioid-free pain control	Time to three or more consecutive days of < 4/10 pain score on a 0–10 NRS with no opioid use for the operated knee		hazard ratios, 95% CI; Kaplan–Meier survival curve
Presence of chronic postsurgical pain (CPSP) at 3, 6, 9 and 12 months	Presence of CPSP as adopted in the International Classification of Diseases (ICD)-11 version by World Health Organization		descriptively as point estimates and 95% CI by group, with minimally important differences presented for context
CPSP pain intensity at rest and movement	Average pain score over the previous week in 0–10 NRS		
Presence of persistent opioid use (POU)	Presence of daily opioid use, started after surgery or increased after surgery (binary)		
Satisfaction with pain control at 3, 6, 9 and 12 months	Using a 0 to 100 scale (0 = extremely dissatisfied, 100 = extremely satisfied)		
Return to function at 3, 6, 9, and 12 months	Using the Return to Function questionnaire		
Knee function at 3, 6, 9 and 12 months	Using Oxford Knee Scale (OKS)		
Quality of Life	Using Euro-Qol 5 Dimensions (EQ-5D) instrument		
Economic analysis	Intervention costs and healthcare resource utilization information as well as information on productivity (e.g. time missed from work) will be collected using a self-administered questionnaire, developed for the purpose of this study		QALYs associated with each study arm will be reported as point estimates along with confidence intervals but not compared in a formal economic evaluation
Complications	Surgery-related and knee-related adverse events, pain medication related adverse events, readmissions, and serious adverse events (SAEs)		descriptively by group

**Table 2:** Feasibility criteria and determining success

Feasibility criterion	Proceed	Proceed with modifications	Do not proceed/major modifications
Adherence (at least 3 of 4 components)	> 90% of participants	80–90% of participants	< 80% of participants
Recruitment	100 participants in 4 months	100 participants in 4–12 months	< 100 recruited or > 12 months
Retention	> 90% of participants	80–90% of participants	< 80% of participants

#### Secondary (clinical) outcomes

The following will be the outcomes for our definitive trial. Pain control and opioid analgesics are interlinked outcomes [43]. Recent studies have highlighted the need to consider both opioid use and pain control as patient-important, and the need to evaluate pain and opioid use trajectories [39, 44]. Hence, our primary outcome for the definitive trial will be to assess the effect of the multicomponent pain management pathway on ‘opioid free pain control’ at 8 weeks after TKA; defined as three or more consecutive days of < 4/10 average pain score on a 0–10 NRS with no opioid use for the operated knee. Secondary outcomes will include presence of CPSP, intensity of resting and movement evoked pain, POU, satisfaction with pain control, quality of life and complication rates. These outcomes will be collected at 3, 6, 9 and 12 months. Selection of our clinical outcomes was guided by the core outcome set developed by Wylde et al. [45] with some modifications. We also considered the need to evaluate outcomes that are patient important. As suggested by Wylde et al., we measure patient reported pain intensity both at rest and with movement. Assessment of CPSP has been considered based on international association for the study of pain definition (IASP) definition, which has been adapted in the International Classification of Diseases-11 version [46]. For evaluating functions, we use the Oxford Knee Score (OKS), which is a 12-item joint-specific questionnaire designed to assess pain and functional limitations in patients undergoing TKA. In a study of 505 patients undergoing primary TKA, OKS showed a good correlation with improvements in OKS in relation to pain (*r* = 0.56; *p* < 0.001) and physician function *r* = 0.56; *p* < 0.001) [47]. EQ-5D is a validated and commonly used tool developed by the EurQol group to measure quality of life. In a study of 758 patients with hip or knee OA, EuroQol (EQ-5D-5L) showed good reliability and internal consistency (Cronbach's alpha was 0.86) along with strong correlation with Western Ontario and McMaster Universities Osteoarthritis Index (WOMAC) pain and function scores (− 0.688 and − 0.782) [48]. To inform the future health economic evaluation of the main trial we will also capture information on the costs of providing the intervention, healthcare resource use, and productivity at 3, 6, 9 and 12 months post-operatively.

#### Measurement of clinical outcomes and economic

Opioid free pain control: For both groups, we will use a daily electronic diary to capture pain scores and opioid use between 1–6 weeks pre-op (to familiarize the patient on the use of the diary) and 8 weeks post-op. We will identify the number of patients achieving opioid-free pain control (defined in previous paragraph) in the intervention and control groups. We have partnered with ManagingLife Inc., who will provide the ManageMyPain app to capture daily pain scores and opioid use. This application is easy to use and secure. It is both Health Insurance Portability and Accountability (HIPPA) and Personal Information Protection and Electronic Documents Act (PIPEDA) compliant and has been recognised by Ontario Health Network (OTN) as an approved platform. This app has been used in previous studies to track pain resolution in surgical patients. Our surgeons estimate that 75% of their patients have a smartphone. For the approximately 25% who do not, we have access to donated smartphones to be used for research purposes. Alternatively for patients who cannot use MMP, a paper diary will be used.

We will measure the presence of CPSP as defined as per the International Classification of Diseases version-11 (ICD-11) [49].

We will measure CPSP Pain Intensity at Rest and during movement using the 0–10 Numerical Rating Scale (NRS).

We will measure persistent opioid use (POU) as a binary outcome as defined as the presence of daily opioid use, started after surgery or increased after surgery.

Using a 0 to 100 scale (0 = extremely dissatisfied, 100 = extremely satisfied), we will measure satisfaction with pain control.

We will assess return to function using the 5-item Return To Function (RTF) questionnaire developed at McMaster University to assess the ability to return to work, home and leisure activities. The RTF questionnaire has been used previously in orthopaedic trials, including an FDA-regulated orthopaedic device trial [50], to determine when a trial participant returns to work, leisure and activities around the home after an injury or surgery.

Using the 12-item Oxford Knee Score, we will assess improvement in knee function and pain following total knee replacement [51].

We will assess health-related quality of life using the Euro-QoL 5 Dimensions instrument consisting of 5 dimensions, including mobility, self-care, usual activities, pain/ discomfort and anxiety/ depression, which contains five levels of answers per dimension [52].

We will collect intervention costs and healthcare resource utilization information (e.g. hospitalization, physician visits) as well as information on productivity (e.g. time missed from work) using a self-administered questionnaire, which we developed for the purpose of this study based on our previous work [53].

We will also collect any surgery-related and knee-related adverse events (AEs), pain medication related adverse events, readmissions and serious adverse events (SAEs). We do not anticipate many risks to study participants beyond usual care. We provide education about safe opioid use and disposal and recognize the potential for opioid withdrawal if opioids are tapered too rapidly. Postoperative prescriptions will be structured and any reduction to chronic opioid use will be monitored, and our pain physicians will develop a structured set of operating procedures to minimize this risk, both preoperatively and postoperatively. Most importantly, the approach throughout the trial will be one of participant engagement and shared decision making. Any patients experiencing AE or SAEs will receive the necessary care at their institution.

### Randomization

The unit of randomization will be individual participants. We will use a 1:1 allocation ratio, stratified by site, with random block sizes of 2 and 4. Randomization will be completed by research personnel, 1–6 weeks before surgery to allow time for the pre-operative education interventions. We will use a centralized online randomization system integrated into REDCap to ensure allocation concealment. A statistician not otherwise associated with the trial will generate the randomization sequence.

### Study follow-up

Participants will be followed from the time of their study inclusion (1–6 weeks pre-surgery) to 12 months after surgery. We will collect baseline data before surgery. We will collect daily pain scores using an electronic diary up to 8 weeks after surgery, and we will collect post-operative outcomes at 3, 6, 9 and 12 months after surgery (Table 3).

**Table 3:** Schedule of EVENTS

Study event	Pre-op 1–6 weeks	In hospital	Postoperative weeks						Months			
			**1**	**2**	**3**	**4**	**6**	**8**	**3**	**6**	**9**	**12**
Screen and consent	X											
Identify high risk patients	I											
Pain education	I											
CBS intervention	I											
Electronic pain and opioid diary	X	X	X	X	X	X	X	X				
Check in with coordinator	I	I	I	I	I	I	I	I				
CPSP assessment^39^, and pain intensity with rest and movement									X	X	X	X
Opioid use	X	X						X	X	X	X	X
Satisfaction with pain control									X	X	X	X
Return to function									X	X	X	X
Knee function	X								X	X	X	X
EQ-5D	X								X	X	X	X
Health economics									X	X	X	X
Complications	X	X	X	X	X	X	X	X	X	X	X	X

*X* all groups, *I* intervention group only

### Protecting against sources of bias

#### Blinding

Due to the nature of the study interventions, participants and the health care team cannot be blinded. We will have an independent blinded surgeon to evaluate each adverse event to minimize the risk of bias for that outcome. The primary study outcome of non-opioid pain control will be collected using a daily e-diary up to 8 weeks. Other study outcomes will be collected by research personnel not involved in the participants’ clinical care. Data analysts will be blinded for all outcomes.

#### Minimizing contamination and co-interventions

Since the existing standard of care does not involve the coordinator or any component of interventions, there is minimal risk of contamination. Patients are allowed to receive other interventions outside of the study, but the role of a pain management coordinator currently does not exist outside of our study. Alternatively, there is a risk if patients are randomised to intervention but do not ultimately receive it. To minimize the risk of crossover, all efforts will be taken to maintain communication between the research personnel and the pain management coordinator. We will also hold weekly team meetings to provide updates on all patients. Participants who are receiving two joint replacements during the study period will only be included for one of their surgeries.

#### Minimizing expertise bias

The CBS sessions are based on the principles of CBT. Although no formal CBT training is required, we will employ pain management coordinators with some prior patient contact experience within the healthcare setting, and we will also develop a pain CBS ‘bootcamp’ to ensure pain management coordinators can successfully implement the CBS sessions, along with training for safe opioid weaning. We also have a pain psychologist within the study team to resolve any challenges relating to CBS sessions for specific participants.

Components of the intervention such as personalized prescriptions, pain management and support may also present expertise bias, as the pain management coordinator will be the participant’s first point of contact for all components. Therefore, to maintain consistency among sites, we will also engage the lead pain physician to liaise with each site to support the pain management coordinator in any decisions regarding pain management and prescriptions.

#### Minimizing attrition bias

Once a participant is enrolled in the trial, every reasonable effort will be made to follow the participant for the entire duration of the study period (12 months). Previously established orthopaedic-specific procedures developed and refined at our central coordination and methods centre will be implemented to improve participant retention. Our research group has consistently used and improved our participant retention strategies over the past 15 years and has published papers on minimizing loss to follow-up in orthopaedic trials [54]. In our group’s four most recent large trials, the loss to follow-up percentages were A-PREP trial–4% [55], HEALTH trial–14.9% [56], FLOW trial–10% [57], FAITH trial–9% [58]. Key strategies used to minimize loss to follow-up: aligning the follow up with standard of care visits; collecting more than one piece of contact information for the participant; research personnel will verify participants’ contact information at each visit and ask the participants their preferred form of contact; prioritizing outcomes if there is participant burden; and requesting permission to access medical records.

### Statistical methods

#### Sample size determination

This pilot trial is not powered to detect clinical differences, so we based our sample size on pilot trial sample size calculations using a confidence interval approach suggested by Thabane et al. [59]. We believe the study will be feasible if participant retention is 90% or greater and will consider > 80% retention acceptable with modifications. If 90/100 participants adhere to the study intervention, then the lower bound of the confidence interval will exclude 80% and we will consider the trial feasible. Therefore, we will include 100 participants in this pilot trial.

#### Primary analysis

Feasibility outcomes will be reported descriptively as numbers and percentages with 95% confidence intervals (CI). This pilot trial will not be powered to detect differences in clinical outcomes. Instead, we will report all clinical outcomes descriptively as point estimates and 95% CI, with minimally important differences presented for context, where available. We will also report hazard ratios for our primary outcome and time-to-event data graphically using a Kaplan–Meier survival curve. We will not impute for missing data in the pilot trial. All analyses will be conducted as intention-to-treat. We will prepare a full statistical analysis plan (SAP) for the definitive trial analysis.

#### Interim analysis

For this pilot trial, data will be analyzed only after completion of data collection. Interim analysis will be considered for the definitive trial.

#### Subgroup and other analyses

Subgroup and other analyses will only be considered for the definitive trial.

#### Economic analyses

To assist with the future economic evaluation of the definitive trial, we will collect information on costs (e.g. intervention costs, costs related to healthcare resource utilization and productivity) and quality of life. Healthcare resource utilization (e.g. hospitalization, emergency department visits, physician visits) and productivity (e.g. time missed from work) will be collected at baseline and at 3, 6, 9 and 12 months using a short economic questionnaire. The time recall will be 3 months (e.g. over the last 3 months, have you been hospitalized?). Healthcare resource utilization and productivity will be costed using publicly available unit costs from Ontario (e.g. Ontario Schedule of Benefits) or from the Canadian Institute for Health Information (e.g. hospitalization costs). Health-related quality of lie will be collected at baseline and at 6 and 12 months using the Euro-Qol 5 Dimensions-5L (EQ-5D-5L), which is a well-validated and widely used quality of life instrument that can assess health utilities for the purpose of health economic analyses. Using the Canadian algorithm of the EQ-5D-5L [60], the health utility scores derived from the EQ-5D-5L questionnaire will be weighted by time spent in health states using an area-under-the-curve approach to calculate quality-adjusted life-years (QALYs). We will also request participants consent for potential data linkage with Institute for Clinical Evaluative Sciences (ICES) administrative data. Since this is a pilot trial, costs and QALYs associated with each study arm will be reported as point estimates along with confidence intervals but not compared in a formal economic evaluation. The analyses will be conducted from the payer (e.g. Ministry of Health) and societal perspectives.

### Data monitoring

#### Steering committee

Our co-investigators make up the Steering Committee for the trial. Steering Committee members are an interdisciplinary group of experts in key fields including anaesthesia/ pain management, orthopaedic surgery, health economics, biostatistics, psychology, pharmacy, occupational therapy, physiotherapy and clinical trials methodology. The Steering Committee will be chaired by the PI and will be responsible for advising on key clinical and methodological issues at all stages of the trial. For the pilot trial, we do not plan to have a formal Data & Safety Monitoring Committee (DSMC) because all interventions are standard care and are not expected to pose greater risk than the control group arm.

#### Trial coordination

The Centre for Evidence-Based Orthopaedics (CEO), McMaster University, will be the trial Methods Centre and will be responsible for coordinating the day-to-day operations of the trial. The CEO has conducted some of the largest multinational trials and observational studies in orthopaedics, including the PREPARE and A-PREP trials (*n* = 8000), FLOW trial (*n* = 2551), the PRAISE study (*n* = 2945) and the INORMUS study (*n* = 30,000). The CEO has the infrastructure to successfully conduct large trials including research coordinators, data managers, statisticians, a network of investigators and required office space.

### Ethical considerations

This study will be conducted according to international standard of ICH-GCP, applicable government regulations and institutional research policies and procedures. We will require ethics approval from each site’s local REB prior to initiating this trial protocol.

### Dissemination policy

While emphasizing the core concepts and delivery involved in the preoperative and postoperative components, our interventional pathway is designed to be adaptable to individual centres. The pre-operative education component will be made available online free of charge. The intervention can also be easily adaptable to other surgical fields. We will partner with the Canadian Orthopaedic Association (COA) and Canadian Anesthesiologists Society (CAS) to help disseminate our study information to orthopaedic surgeons and anesthesiologists in Canada, plus international members. We will also partner with our university and hospital press offices to distribute a press release for the general public.

## Data Availability

Not applicable.

## Abbreviations

AE: Adverse event
CAS: Canadian Anesthesiologists’ Society
CBS: Cognitive behaviour skill sessions
CBT: Cognitive behavioural therapy
CEO: Center for Evidence-Based Orthopaedics
CI: Confidence interval
COA: Canadian Orthopaedic Association
CPSP: Chronic post-surgical pain
CRF: Case report form
DSMC: Data Safety and Monitoring Committee
EDC: Electronic data capture
ERAS: Enhanced recovery after surgery
EQ-5D-5L: EuroQol 5 Dimensions
HIPPA: Health Insurance Portability and Accountability Act
ICES: Institute for Clinical and Evaluative Sciences
KT: Knowledge translation
MED: Morphine equivalent dose
NRS: Numerical rating scale
NSAID: Non-steroidal anti-inflammatory drug
OA: Osteoarthritis
OREOS: Opioid reduction and enhanced recovery in orthopaedic surgery
OTN: Ontario Telehealth Network
PIPEDA: Personal Information Protection and Electronic Documents Act
POU: Persistent opioid use
PSH: Perioperative surgical home
QALY: Quality-adjusted life-year
RCT: Randomised controlled trial
REB: Research ethics board
SAE: Serious adverse events
SAP: Statistical analysis plan
TKA: Total knee arthroplasty
